# Microgravity and evasion of plant innate immunity by human bacterial pathogens

**DOI:** 10.1038/s41526-023-00323-x

**Published:** 2023-09-07

**Authors:** Noah Totsline, Kalmia E. Kniel, Harsh P. Bais

**Affiliations:** 1 Department of Plant and Soil Sciences, AP Biopharma, University of Delaware, Newark, DE USA; 2 Department of Animal and Food Sciences, University of Delaware, Newark, DE USA

**Keywords:** Microbiology, Plant sciences

## Abstract

Spaceflight microgravity and modeled-microgravity analogs (MMA) broadly alter gene expression and physiology in both pathogens and plants. Research elucidating plant and bacterial responses to normal gravity or microgravity has shown the involvement of both physiological and molecular mechanisms. Under true and simulated microgravity, plants display differential expression of pathogen-defense genes while human bacterial pathogens exhibit increased virulence, antibiotic resistance, stress tolerance, and reduced LD_50_ in animal hosts. Human bacterial pathogens including *Salmonella enterica* and *E. coli* act as cross-kingdom foodborne pathogens by evading and suppressing the innate immunity of plants for colonization of intracellular spaces. It is unknown if evasion and colonization of plants by human pathogens occurs under microgravity and if there is increased infection capability as demonstrated using animal hosts. Understanding the relationship between microgravity, plant immunity, and human pathogens could prevent potentially deadly outbreaks of foodborne disease during spaceflight. This review will summarize (1) alterations to the virulency of human pathogens under microgravity and MMA, (2) alterations to plant physiology and gene expression under microgravity and MMA, (3) suppression and evasion of plant immunity by human pathogens under normal gravity, (4) studies of plant-microbe interactions under microgravity and MMA. A conclusion suggests future study of interactions between plants and human pathogens under microgravity is beneficial to human safety, and an investment in humanity’s long and short-term space travel goals.

## Introduction

Plants have been successfully cultivated and consumed in space, a fact surprising to those unfamiliar with the field of astrobotany. As humans venture deeper into space for greater amounts of time, human nutritional health and food safety during spaceflight are increasingly important. Nutrition provided by space-grown crops could mitigate human health risks associated with spaceflight^1,2^ including reductions in bone density, reduced eye health, poor access to vitamin D, reduced uptake of calcium, oxidative stress via cosmic radiation, increased anxiety, and depression, and immunosuppression associated with reduced T cell and natural killer cell levels^3–5^. Leafy green vegetables are ideal candidates for spaceflight cultivation as they are nutritionally dense and require less space especially when grown as microgreens^3,6^. Microgreens offer an alternative to pre-packaged meals delivered to the crew of the International Space Station (ISS) that degrade in nutritional content overtime, especially in labile vitamins B1, A, and C, and are resource intensive in terms of payload delivery^7^. However, given the diverse microbiome including foodborne pathogens aboard the International Space Station (ISS), there is risk of crop contamination^8,9^. On Earth, produce contamination by bacterial pathogens is a regular cause of foodborne disease and fruits and vegetables are contaminated in both pre- and post-harvest conditions by human enteric pathogens^10^. Increasing evidence suggests foodborne human-pathogenic bacteria use mechanisms employed by phytopathogens to suppress and evade plant innate-immunity to colonize plant intracellular-spaces while maintaining their mammalian virulency potential^11,12^ (See Table 1 for the interaction of human pathogens with plants). Despite stringent pre-launch biosafety precautions, the survival and persistence of multiple genera of enteric human pathogens have been documented on multiple surfaces throughout the International Space Station (ISS), including foodborne bacterial pathogens such as *Salmonella enterica*, *Staphylococcus aureus*, *Escherichia coli*, and *Shigella sonnei*^9,13^. Although no human pathogens have been recovered from space-grown produce so far, a diverse group of fungal and bacterial genera have colonized the leaf and root tissue of space-grown lettuce, including genera containing human pathogens (*Staphylococcus*, *Pseudomonas*, *Aspergillus*), suggesting potential for crop contamination^1^. Space vehicles represent evolutionarily novel ecosystems in which humans, the microbiome, and plants are exposed to a microgravity environment lacking in physical forces of weight, buoyancy, convection, and shear^14,15^. Numerous studies demonstrate that microgravity increases antibiotic resistance^9,16,17^, virulency^18–21^, and stress tolerance^22–25^ in human pathogenic bacteria. Studies suggest microgravity increases plant susceptibility to fungal phytopathogens^26–29^, and yields differential expression of pathogen-related plant genes^30,31^. However, the relationship between plants, human pathogens, and microgravity is critically understudied, and studies regarding plant interactions with bacteria under microgravity in general are lacking. Taken together, it is imperative that interactions between plants and human pathogens under microgravity be examined to ensure spaceflight food safety and as an investment in humanity’s long-term space travel goals.

**Table 1:** Examples of opportunistic human bacterial pathogens infecting plant hosts.

Salmonella enterica serovar Typhimurium	Arabidopsis thaliana, Brassicaceae Tomato (Solanum lycopersicum), Solanaceae Tobacco (Nicotiana tabacum), Solanaceae Lettuce (Lactuca sativa), Asteraceae	^90^ ^108^ ^94^ ^12^
Shigella spp.	Arabidopsis thaliana, Brassicaceae	^92^
Escherichia coli O157:H7	Spinach (Spinacia oleracea), Amaranthaceae Lettuce (Lactuca sativa), Asteraceae Arabidopsis thaliana, Brassicaceae	^109^ ^110^
Pseudomonas aeruginosa	Lettuce (Lactuca sativa), Asteraceae Arabidopsis thaliana, Brassicaceae	^89^
Staphylococcus aureus Enterococcus faecalis	Arabidopsis thaliana, Brassicaceae Arabidopsis thaliana, Brassicaceae	^88^ ^123^

## Human bacterial pathogens in microgravity

### Pathogen virulence is altered under microgravity

Given the persistence of human bacterial pathogens aboard the International Space Station^9,13^, understanding pathogen behavior under microgravity is necessary to protect human spaceflight safety. Human bacterial pathogens display a wide range of altered behaviors associated with increased pathogenesis under spaceflight and modeled microgravity analogs (MMA). These include higher cell counts^15^, increased growth rate^23,32^, increased biofilm formation^16^, cell aggregation^20,32^, altered motility and chemotaxis^33^, increased expression of Type-III Secretion System (T3SS) related pathogenicity island genes^34,35^, altered virulency in animal hosts^18–21^, increased resistance to hydrogen peroxide^36^, increased macrophage survivability^20,21,23^, increased resistance to antibiotics^9,16,17^, increased tolerance to acidic conditions^21^, and reduced LD_50_ in mice^20,21,23^. *Salmonella* Typhimurium cultured under MMA was more lethal compared to normal-gravity culture in mice with a 5.2-fold decrease in LD_50_ and shorter average time to death^21^. Spaceflight-cultured *Salmonella enterica* Typhimurium (henceforth, *Salmonella* Typhimurium) exhibited decreases in LD_50_ as low as 6.9-fold in mice compared to normal gravity bacterial cultures^37^. Co-culture of *Salmonella* Typhimurium and functional macrophages in a biomimetic 3-D model of human colonic epithelial tissue exhibited increased host colonization and survival inside macrophages under MMA compared to normal gravity^35^, potentially due to increased acid tolerance. While a wide range of altered pathogen behaviors under spaceflight and MMA have been documented, the biophysical and molecular drivers of these alterations are only recently being uncovered.

### Low fluid shear modulates stress tolerance and virulency in human pathogens

Bacterial pathogens must survive in a diverse set of ecological niches both within and outside the host. This requires pathogens to sense and respond to a variety of environmental stresses. Responses are complex, occurring at the transcriptomic and post-transcriptomic level, involving interrelated regulatory networks behaving synergistically and antagonistically^38^. Low fluid shear is an initial biophysical stimulus implicated in recent studies as the source of enhanced stress tolerance and virulency observed in human pathogens under microgravity/MMA^22,34^ and within low-fluid shear microsites (>1 dyne/cm^−2^) of the human lumen, brush border microvilli, respiratory system, and urogenital tracts during normal pathogenesis^37,39^. Low fluid-shear in spaceflight/MMA has been observed to increase acid tolerance and virulence factor gene expression in *Salmonella* Typhimurium and *E. coli* O157:H7^21,24,32,36^, increase stress tolerance, antibiotic resistance, and biofilm formation in *E. coli* O83:H1^25,40^, and induce peak expression of *E. coli* O157:H7 locus of enterocyte effacement (LEE) pathogenicity island responsible for attachment to host epithelial cells and formation of lesions^34^. Adaptation to low-fluid shear conditions within the human gastrointestinal tract during normal disease progression in human hosts may predispose enteric bacterial pathogens to tolerance of similar conditions found in spaceflight/MMA.

### Altered extracellular transport modulates virulence phenotypes under low fluid shear

Theoretical models suggest alterations to the intracellular processes of bacteria by gravity are unlikely given the scale of microorganisms^41^. The altered extracellular environment model posits reduced bulk transport of nutrients by bacteria from the extracellular environment due to a lack of convection in spaceflight is the primary source of all microgravity phenotypes^15,32^ Acres et al., 2021). Reduced uptake of phosphate and oxygen in spaceflight has been linked to enhanced virulency of *Salmonella* Typhimurium^37^ and higher final cell counts of *Pseudomonas aeruginosa*^42^. Low oxygen and phosphate are host signals for enhanced expression of virulence phenotypes in enteric pathogens within the human gastrointestinal tract under normal gravity^43–45^. On the other hand, bacterial scavenging of iron from the extracellular environment appears enhanced under microgravity. Upregulation of iron metabolism genes and the Hfq-regulated gene *fur* encoding the membrane bound Ferric Uptake Regulator Protein (Fur) were observed in both spaceflight and MMA cultured *Salmonella* Typhimurium conferring increased acid tolerance^23,37^, an important virulence factor in enteric pathogens^46^. Iron is essential for virulence in many enteric pathogens^47^. Anerobic conditions like the host environment of the human gastrointestinal tract were associated with upregulation of virulence genes controlled by *fur* in *Salmonella* Typhimurium in a normal gravity experiment^47^. Interactions at the liquid media and cell envelope interface are subject to altered physical forces under microgravity, modulating both active and inactive forms of nutrient transport which contribute to altered virulency and stress tolerance. This may be attributed to an overlap between environmental signals triggering expression of virulence genes encountered by enteric pathogens in both spaceflight conditions and during disease progression within the human body under normal gravity. Altered access to oxygen, phosphate, and iron specifically appear to be drivers of enhanced virulence phenotypes observed in spaceflight cultures of enteric pathogens.

### Molecular basis of altered virulence of human pathogens under microgravity

A variety of functionally diverse genes related to metabolism, stress tolerance, and virulency are differentially expressed under microgravity and MMA compared to normal gravity in human pathogenic bacteria^15,24,39^. However, meta-analyses have questioned the existence of a universal bacterial response to microgravity due to studies being conducted using a variety of media compositions, viscosities, temperatures, spaceflight or MMA hardware, and bacterial species^48^. On the other hand, decreased expression of the post-transcriptional global regulator Hfq in spaceflight/MMA has been consistently observed across studies of multiple pathogens^33^ including Gram-negatives *Salmonella* Typhimurium^20^, *Pseudomonas aeruginosa*^49^ and the Gram-positive *Staphylococcus aureus*^16^ representing the first differentially expressed spaceflight regulon common to multiple bacterial species. Hfq is an sRNA and mRNA binding protein conserved in many bacterial species which positively and negatively regulates expression of a wide range of stress response and virulency genes in bacterial pathogens of animals and plants^50^ (See Table 1 for interaction of human pathogens with plants). Spaceflight microgravity and MMA represent powerful environmental signals producing global differential expression of genes and altered phenotypes, often contributing to increased virulency and stress tolerance. More studies are needed to further elucidate the role of Hfq as a spaceflight/MMA regulon contributing to altered virulence.

## Plants in microgravity

### Plant gravitropism

**Fig. 1:**
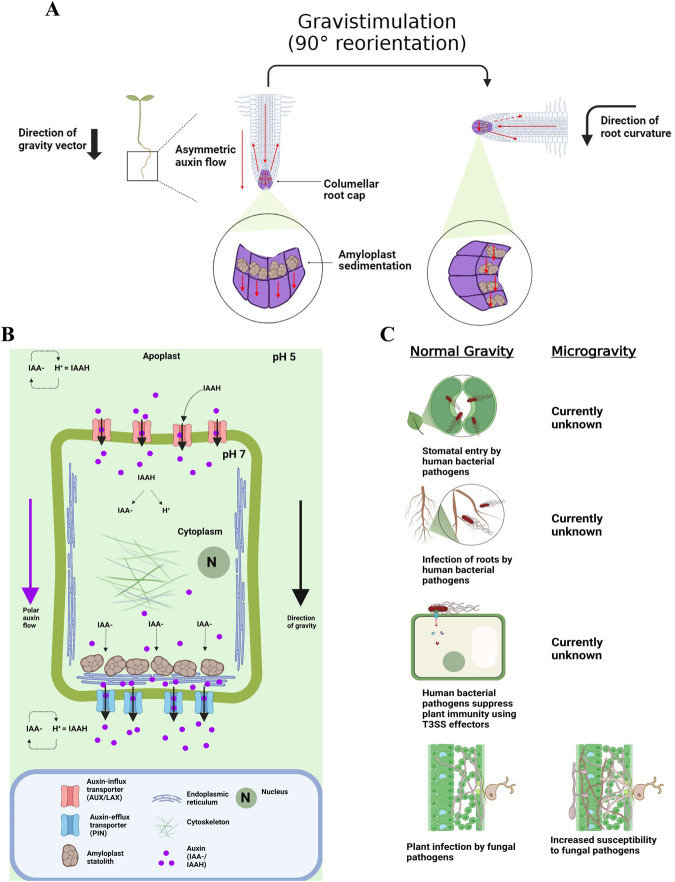
Comparison of root and foliar response to microgravity and pathogen ingression. **A** Roots are positively gravitropic and grow towards gravity. Growth is directed by asymmetric redistribution of the plant growth hormone auxin. **B** The initial biophysical stimulus of gravitropic phenotypes is thought to be sedimentation of amyloplast at the bottom statocyte cells. Auxin influx/efflux proteins mediate polar auxin flow. **C** Under normal gravity human bacterial pathogens suppress and evade plant immunity to enter plant apoplastic space through foliar stomata and roots. Plant interactions with human pathogens under microgravity are unknown. Furthermore, plant responses to microgravity at the foliar level are poorly elucidated in addition to plant interactions with bacteria under microgravity in general. Studies demonstrate an increased susceptibility to fungal pathogens under microgravity.

Like all known life, plants have evolved in response to Earth’s gravity. Land plants have evolved roots which grow downward towards gravity (positive gravitropism), and shoots which grow upward away from gravity (negative gravitropism)^51^. Gravitropism generally occurs in three sequential phases: biophysical signal perception, signal transduction, and directed growth. Plant perception of the gravity vector (gravisensing) occurs in specialized cells known as statocytes which are present in the columella cells of the root tip and in the shoot endodermis^52^. The leading hypotheses for plant gravisensing are the starch-statolith sedimentation model and tensegrity model. Both models are not mutually exclusive and could contribute for gravisensing in tandem^52^. The starch-statolith model proposes dense, starch-based leucoplasts called amyloplasts provide the initial biophysical signal of gravisensing via sedimentation at the bottom of statocyte in accordance with the gravity vector^51^. The tensegrity model emphasizes deformation of the actin-based cytoskeletal mesh within the statocyte as the initial biophysical signal of gravisensing^51^ (See Fig. 1A, B for root gravisensing). Following an initial stimulus, signal transduction transmits a signal to distally located regions of the plant^52^. The divalent calcium cation Ca^2+^ is a common messenger in many plants signaling pathways and is thought to be the chemical signal of gravitropism, potentially modulated by inositol trisphosphate (InsP3)^52,53^. Directional growth occurs via asymmetric redistribution of the plant hormone indole-3-acetic acid (auxin/IAA), as first described in the Cholodny-Went model^54^. Auxin redistribution is facilitated by transmembrane efflux transporter proteins of the PIN family and influx transmembrane proteins of the AUX/LAX family^55^. The polar localization of these transporters within the statocyte cell allows for directional movement of auxin to distal plant tissue^55^ (See Fig. 1A, B). The pH-dependent movement potential of protonated auxin (IAAH) and deprotonated auxin (IAA^-^) also facilitates its transport, with only IAAH capable of transmembrane diffusion^52^ (See Fig. 1A, B for gravisensing).

### Plant responses to microgravity

Plants grown under true or simulated microgravity display altered physiology and gene expression. Plant responses to microgravity/MMA display a great degree of variability amongst plant species and cultivars^56^. Physiological responses to microgravity include spontaneous curvature of roots and shoots^57,58^, elongation of rice coleoptiles^59^ and hypocotyls^60^, increased growth rate and length of inflorescence stems^57^, and reduced overall yield^2,56^. Impaired biogenesis of the cell wall polymers lignin, cellulose, and hemicellulose have been reported under microgravity in roots, hypocotyls, and protoplast^61–63^. Altered cell wall phenotypes under microgravity have been attributed to differential expression expansin and extensin genes responsible for cell wall loosening observed in both spaceflight^64^ and MMA^63^, decreased activity of cell-wall-bound peroxidases involved in lignin synthesis, and rearrangement of cortical microtubules which direct cellulose synthase complexes and orientation of cellulose microfibrils^61,63,64^. A functionally diverse group of differentially expressed genes (DEGs) occur in response to microgravity/MMA including those associated with reactive oxygen species (ROS)^65,66^, antioxidant enzymes^66^, auxin transport^64,67,68^, cytoskeletal modification^69^, cell-wall development^64,69^, Ca^2+^ messenger molecules^64,66,68^, mitogen-activated protein kinase (MAPK) cascade signaling^70^, cytokinin signaling^68^, RuBisCO gene expression^66^, carbohydrate metabolism^67^, DNA repair^69^, and differential expression of pathogen-defense genes^69^. Nearly half of all upregulated DEGs in *Arabidopsis* seedings exposed to spaceflight were related to wounding and pathogen-defense^30^, while another spaceflight study using *Arabidopsis* found a PR-1-like gene to be the most downregulated DEG compared to normal gravity control^64^. Exposure to microgravity has been proposed as comparable to pathogen exposure in terms of DEG overlap^31^. Future studies exposing plants to pathogens in microgravity conditions could differentiate DEG responses unique to microgravity as compared to pathogen infection and elucidate the relationships between gravity and plant immunity.

## Plant immunity against pathogens

### Induced immunity in plants

Unlike the immune system of vertebrate animals, plant immunity is non-adaptive, relying instead on the innate immune capabilities of each plant cell. Plants have two distinct forms of inducible immunity to pathogens known as systemic acquired resistance (SAR) and induced systemic resistance (ISR)^71^. Once properly stimulated, SAR and ISR provide systemic transcriptional reprogramming in uninfected tissues throughout the whole plant, priming them for heightened defense. Primed plants display faster and stronger expression of plant-defense genes following subsequent pathogen exposure and decreased disease severity compared to unprimed plants^72^. SAR is mediated by the plant hormone salicylic acid (SA), while ISR is mediated by the plant hormones jasmonic acid (JA) and ethylene^71^. SAR is activated by biotrophic and hemibiotrophic pathogens which receive nutrition from living cells but can also be activated by avirulent or nonpathogenic microbes. In contrast, ISR is activated by necrotrophic pathogens which feed upon dead host tissues and is also activated by beneficial microorganisms which colonize the plant root system and the root-soil interface known as the rhizosphere^71^. SAR and ISR inhibit the response of the other due to the antagonistic relationship between their respective signaling hormones SA and JA^73^. For plant immunity to be induced, plants must successfully perceive the invading pathogen.

### Pattern-triggered immunity in plants

In pattern-triggered immunity (PTI), plants recognize distinctive features carried by pathogens to initiate a defense response. PTI is a broad defense response against entire classes of pathogens following recognition of pathogen-associated-molecular-patterns (PAMPs) by corresponding pattern recognition receptors (PRRs) located on the surface of plant cells^74^. PAMPs are small molecular motifs conserved in a class of microorganisms which do not occur within the host. PAMPs include the flagellar peptide flg-22, lipopolysaccharides (LPS), peptidoglycan, chitin, the bacterial elongation factor EF-Tu, elf18 peptide, and β-glucan^75^. PRRs are transmembrane multiprotein complexes that exist as either receptor-like kinases (RLKs) or receptor-like proteins (RLPs)^76^. RLKs consist of a ligand-binding ectodomain, a transmembrane domain, and an intracellular kinase signaling-domain. RLPs differ from RLKs in a lack of an intracellular kinase domain or any other intracellular signaling domain. The ectodomains of PRRs vary in composition and determine the range of PAMPs which may bind, and initiate defense signal transduction required to trigger PTI. Common PRR ectodomains are leucine-rich repeat (LRR) domains and lysin motif (LysM) domains, although others exist^76^. Once initiated, PTI confers production of reactive oxygen species (ROS), stomatal closure, callose deposition at cell walls, shifts in apoplastic pH, production of anti-microbial secondary metabolites, polymer-degrading enzymes such as chitinase, expression of pathogenesis-related genes (PR genes), and activation of mitogen-activated protein kinase (MAPK) cascades and SA signaling pathways required to activate SAR^77–79^. A successful pathogen must avoid or suppress these defenses to establish itself within a host.

### Effectors proteins of bacterial pathogens

Through a coevolutionary arms race between pathogen and host, bacterial pathogens have evolved secretory systems to deliver virulence factors known as effector proteins to host tissues^79^. Effector proteins (henceforth, effectors) manipulate host physiology and immunity through highly diverse biological mechanisms of action for promotion of pathogen colonization and virulency^80^. The best studied effectors are those delivered by Gram-negative bacteria which use a needle-like Type-III Secretion System (T3SS) to translocate effectors into the plant apoplast or cytoplasm^81^. Gram-positive bacteria lack a T3SS and instead utilize modified versions of the Sec (general secretory) and Tat (twin-arginine translocation) pathways also found in Gram-negatives to deliver effectors^82^. Effectors provide differing functions based upon the lifestyle and nutritional requirements of a pathogen. Effectors of necrotic pathogens can act directly as toxins killing host tissues, while biotrophic and hemibiotrophic effectors provide evasion or suppression of immunity to allow pathogens to remain undetected within the host^83^. It should be noted, plant pathogen lifestyle and host niche are complex and there is functional overlap of effectors along the necrotrophic to biotrophic pathogen continuum, with some necrotrophic pathogens possessing cryptic biotrophic phases and biotrophs displaying necrotrophic phases^83^. By suppressing or evading inducible plant immunity through a variety of mechanisms, effector proteins cause effector-triggered susceptibility (ETS), enabling infection in a plant host^84^. Plant hormones are common targets for manipulation by effectors due to their role in SAR/ISR defense signaling^80^. Transcription factors regulating JA are targeted by numerous effectors in plant pathogens demonstrating the importance of hormone manipulation in successful virulency^85^. By mimicking the structure of JA or triggering JA transcription factors, effectors exploit the antagonistic relationship between JA and SA to suppress SAR signaling and stomatal closure^73^. Effectors also manipulate host gene expression to suppress immunity or create a more habitable host environment by regulating activity of endogenous transcription factors or by acting directly as transcription factors, as exemplified by transcription activator-like effectors (TALEs) in pathogenic *Xanthomonas* and *Ralstonia*^80^.

### Effector-triggered immunity in plants

In response to effectors, plants have evolved intracellular receptors to recognize effectors and activate effector-triggered immunity (ETI). ETI involves initiation of MAPK cascades and expression of pathogenesis-related proteins (PRs) like PTI^74^. Both pathways also trigger systemic acquired resistance (SAR), mediated by salicylic acid (SA) associated signaling elements, triggering transcriptional reprogramming in uninfected distally located plant organs, priming them for defense^75^. In contrast, ETI is a faster and more powerful localized hypersensitive response compared to PTI^74^. To recognize effectors and initiate ETI, plants have evolved resistance genes (R genes) encoding R proteins within the cytoplasm. R proteins typically containing a nucleotide-binding site leucine-rich repeat (NBS-LRR) domain to bind effectors. The LRR domain can exist alongside other domains including toll-interleukin receptor (TIR), coiled-coil (CC), or WRKY, in addition to existing in multiples, or less commonly being absent all together^86^. R proteins can bind directly to effectors to trigger ETI or act as “guard proteins” which recognize structural changes in host proteins targeted by effectors and use these modified host proteins as indirect signals of pathogen effectors^87^. ETI provides a powerful, localized hypersensitive response causing programmed cell death, characteristic formation of necrotic lesions, oxidative bursts, and changes in extracellular pH. This localized hypersensitive response serves to limit the spread of a pathogen beyond the direct site of infection^75^. Effectors of successful pathogens evade or suppress ETI allowing for infection, known as effector-triggered susceptibility (ETS)^79^. Pathogens must circumvent either PTI or both PTI and ETI to successfully invade and proliferate within a plant.

## Plant as hosts to human pathogens

Despite the wide evolutionary distance between plants and humans, numerous pathogens of humans including *Salmonella enterica*, *Pseudomonas aeruginosa*, *Staphylococcus aureus*, *Escherichia coli* O157:H7, and *Shigella* spp. display cross-kingdom pathogenicity by infecting and colonizing both plant and human hosts^11,88–92^ (See Table 1). These pathogens, while typically associated with animal disease, can in fact persist on the surface of plants as epiphytes and *in planta* as endophytes, finding a sheltered and nutrient rich habitat within the apoplast^93^. Furthermore, human pathogens trigger, suppress, and evade plant innate-immunity using virulence factors found in bona-fide plant pathogens^12,91,94^. With some exceptions^95,96^, human enteric pathogens are not true phytopathogens as they do not display prominent signs or symptoms of disease within a plant host^97^. Gram-negative phytopathogens of the genera *Xanthomonas*, *Pseudomonas*, and *Erwinia* use T3SSs to deliver effector proteins to suppress host plant immunity^98^ and new research is revealing similar function of effectors in human pathogens^90,92^ (See Table 1). Interestingly, the post-transcriptomic regulator Hfq implicated in altered virulency of human pathogens under microgravity controls virulency genes in both plant pathogens^99^ and in human pathogens^50^. Human pathogens use overlapping behaviors and virulence factors to infect both plants and humans, reflecting evolutionarily conserved strategies for infection of hosts in evolutionarily disparate eukaryotic kingdoms^95^. Establishment of host-attachment is essential for virulency by enteric pathogens in both animal and plant hosts^93,97^. Plant-host attachment is facilitated by a variety of virulence factors including fimbriae, flagella, type IV pili, bacterial cellulose, and the O-antigen capsule of Gram-negative bacteria^100,101^ all of which have similar function in attachment and subsequent infection of the human gastrointestinal system^102,103^. Biofilms comprised of extrapolymeric substances (EPS) allow enteric pathogens to adhere to and colonize the leaf phyllosphere epiphytically, while providing additional protection from ultraviolet radiation, desiccation, and loss of attachment^101^. To transition from an epiphytic to endophytic lifestyle within the apoplast, bacteria must ingress via physical openings in the plant host^104^. Unlike some fungal and bacterial phytopathogens, human-pathogenic bacteria including *Salmonella* spp. and *E. coli* are incapable of degrading cellulose and must rely upon pre-existing openings in plant tissue for physical entry^105^. Pathogens enter the plant apoplast via roots^106^, wounds^107^, trichomes^93^, hydathodes^105,108^, and stomatal apertures^12,105^. Stomata are particularly important routes of invasion for persistence within the apoplast by *Salmonella* and *E. coli*^93,109^. Populations of *Salmonella* and *E. coli* persist within the apoplast at medically significant levels^110^. Enteric pathogens are also active members of the root rhizosphere microbial community, competing with indigenous bacteria for nutrients and colonization space on the rhizoplane^111^. Bacteria likely ingress roots via epidermal cracks in newly formed lateral roots^105^. To successfully ingress the apoplast and survive *in planta*, pathogens must evade the host plant immune response.

### Human enteric pathogens trigger, evade, and suppress plant innate immunity

Like traditional phytopathogens, it is clear human enteric pathogens trigger PTI and ETI^12,91,112^. The flagellar peptide Flg-22 is a well-characterized PAMP in many bacterial pathogens, and Flg-22 in *Salmonella enterica* is recognized as an epitope by the PRR FLS2 in the model plant *Arabidopsis thaliana*, eliciting a PTI defense response^113^. Purified LPS of *Salmonella* Typhimurium were also found to act a PAMP, triggering PTI in *Nicotiana tabacum*^94^. *E. coli* was also found to induce PTI in both *Arabidopsis* and lettuce^110^. Increasing evidence suggests some human foodborne pathogens suppress and evade plant innate immunity^11,12^ (See Table 1). The Gram-negatives *Salmonella enterica* and *Shigella* spp. use Type-III Secretion System (T3SS) delivered effector proteins to infect and suppress immunity in plant hosts^90,92^. *Salmonella* virulency genes are clustered on two Salmonella Pathogenicity Islands (SPI_1_ and SPI_2_), with each SPI coding for its own T3SS (T3SS-1 and T3SS-2), capable of delivering at least 28 characterized effector proteins in the case of the T3SS-2 of Salmonella *enterica* serovar Typhimurium^114^. *Salmonella enterica* serovar Typhimurium was able to suppress stomatal closure of lettuce using a T3SS-dependent mechanism^12^. In another study, *Arabidopsis* challenged with a *S. enterica* mutant Δ*prgH* lacking a functional T3SS-1 elicited stronger host transcription of genes associated with stress and immunity compared to the wild type, indicating a role of T3SS in host immune suppression^113^. The T3SS-dependent delivery of *S. enterica* effector SpvC encoded on the *Salmonella* plasmid virulence (*spv*) locus was found to dephosphorylate multiple mitogen-activated protein kinases (MPK3, MPK4, MPK6) in *Arabidopsis*, inhibiting PTI^90^. A Δ*spvC* mutant pathogen displayed decreased proliferation *in planta*. Additional *S. enterica* effector proteins were found to trigger a hypersensitive response in *Arabidopsis* leaves^92^. *S. enterica* Δ*InvA* mutants lacking a functional T3SS-1 were reduced in ability to suppress PTI in *Nicotiana tabacum* resulting in stronger oxidative bursts and cytoplasmic pH shifts compared to wild type^94^, indicating a role of effector proteins in immune suppression. *S. enterica* serovar Senftenberg and *S. enterica* serovar Typhimurium exhibit heterogeneity in Flg-22 sequence, thus avoiding PAMP recognition and PTI in an apparent avoidance strategy^115^. Multiple species of *Shigella* (*Shigella boydii, Shigella sonnei, Shigella flexneri*) were found to require T3SS-delivered effectors OspF and OspG to invade and proliferate within *Arabidopsis*^92^. Shigatoxigenic *E. coli* (STEC) O157:H7 was also found to depend upon the T3SS for successful internalization of spinach leaves^109^.

## Plant-microbe interactions under microgravity

**Fig. 2:**
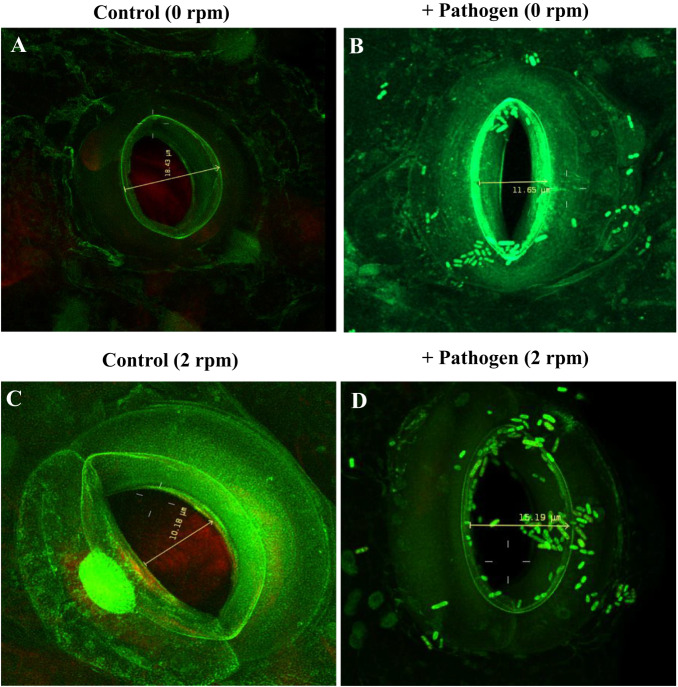
Stomatal physiology in lettuce plants gravistimulated via continuous rotation at 2 RPM. The rod-shaped bacteria seen on the guard cells are *Salmonella enterica* serovar Typhimurium GFP-labelled strain 14028 s. Images acquired 3 hours following ±rotation or ±foliar-treatment with bacteria. **A** Stomate of an unrotated plant without treatment with bacteria. The stomatal aperture appears fully opened. **B** Stomate of an unrotated plant with foliar application of bacteria. Bacteria have prevented complete stomatal closure and began entry to the plant apoplast via the stomatal aperture. **C** Stomate of a rotated plant without treatment with bacteria. The stomatal aperture appears more constricted compared to the unrotated control. **D** Stomate of a rotated plant with foliar application of bacteria. Defensive closure of the stomate appears suppressed to a greater degree than in **B** displaying unrotated plants with bacteria. More bacteria are visible entering the aperture and navigating to a greater depth within the stomatal cavity compared to unrotated plants with bacteria.

Few studies have examined plant-microbe interactions under microgravity and to date, the majority of documented cases involve plant–fungal interactions^26,27,29^ (See Table 2). Increased susceptibility could be attributed to novel microgravity stressors including lack of fluid and gas convection, and buildup of CO_2_ and the gaseous plant hormone ethylene^116^. Impaired synthesis of lignan and cellulose under microgravity could also contribute to susceptibility^63^. Wheat seedlings grown under MMA were more susceptible to the fungal pathogen *Fusarium graminearum*, and the biocontrol properties of *Pseudochrobactrum kiredjianiae* A4 were diminished^28^. *Fusarium oxysporum* also acted as opportunistic fungal pathogen on *Zinnia hybridia* grown aboard the International Space Station, possibly due to increased water stress^29^. In another study, soybean roots were more susceptible to the soybean root rot pathogen *Phytophthora sojae* in spaceflight compared to a ground control, displaying more disease symptoms, higher root colonization, and elevated ethylene levels^26^. Wheat seedlings germinated in spaceflight experienced serious disease from an opportunistic seedborne fungal endophyte of the genus *Neotyphodium* that seedlings germinated on Earth were resistant to^117^. Spaceflight microgravity also significantly altered the endophytic bacterial community of wheat seedlings (*Triticum aestivum*) with a significant shift toward members of family Enterobacteriaceae, while no significant community changes were observed in the rhizosphere^118^. A similar studying using wheat seedlings under MMA found endophytic diversity to increase in the leaf and decrease in the roots^119^. Under spaceflight microgravity, the plant-beneficial bacteria *Rhizobium leguminosarum* bv. *trifolii* displayed enhanced binding to succinate and its synthetic structural analog acetylsalicylic acid (Aspirin), the former being an organic acid synthesized by leguminous plants to sustain *Rhizobium* as endosymbiotic bacteroids which occupy root nodules for nitrogen fixation^120,121^. A tripartite symbiosis study culturing the legume *Medicago truncatula* under continuous MMA alongside either the nitrogen-fixing symbiotic bacteria *Sinorhizobium meliloti*, the arbuscular mycorrhizal fungus *Rhizophagus irregularis*, or both, found reduced overall plant biomass and root nodulation by *S. meliloti* alone, enhancements to plant biomass identical to normal gravity by *R. irregularis* alone, and a slight attenuation to the negative influence of *S. melioti* and MMA on plant biomass with a co-inoculation^122,123^. Overall, these studies suggest microgravity increases plant susceptibility to fungal pathogens, alters plant relationships to microbial symbionts, and modifies community structure of endophytes (See Table 2). The knowledge pertaining to how opportunistic pathogens modulate plant physiology (stomatal opening/closing) under altered gravity conditions is limited. Our work showed that lettuce plants subjected to gravity stimulation via continuous rotation displayed wider stomatal apertures compared to unrotated plants (See Fig. 2). In addition, plants treated with *Salmonella enterica* serovar Typhimurium on foliar surfaces and subjected to rotation showed increased stomatal apertures compared to *Salmonella*-treated unrotated plants (See Fig. 2). Our data suggest that some human pathogens may override plant physiological defense response under microgravity conditions to invade and colonize the apoplast. However, additional studies are needed to elucidate plant susceptibility to bacterial pathogens under microgravity.

**Table 2:** Examples of microbes infecting plant hosts under true or simulated^*^ microgravity.

Fusarium graminearum (fungus)^*^	Wheat (Triticum aestivum), Poaceae	^28^
Fusarium oxysporum (fungus)	Zinnia hybrida, Asteraceae	^29^
Phytophthora sojae (oomycete)	Soybean (Glycine max), Fabaceae	^26^
Neotyphodium sp. (fungus)	Wheat (Triticum aestivum), Poaceae	^117^
Sinorhizobium meliloti (bacteria)^*^	Medicago truncatula, Fabaceae	^122^
Rhizophagus irregularis (fungus)^*^	Medicago truncatula, Fabaceae	^122^

Organisms marked with * were part of modeled microgravity analog (MMA) studies as opposed to spaceflight.

## Concluding remarks

Spaceflight cultivation of plants is essential for maintaining human health during long-term space travel. Human pathogens suppress and evade plant PTI and ETI to colonize and proliferate *in planta*, allowing them to persist as foodborne pathogens. Under microgravity, biophysical and molecular mechanisms such as low-fluid shear and the differential expression of the post-transcriptional global regulator Hfq cause human pathogens to display increased growth rate, resistance to stress and anti-biotics, and increased virulence in animals. While plants have been shown to be more susceptible to fungal and oomycete phytopathogens under microgravity, their susceptibility to colonization by bacterial pathogens including human bacterial pathogens under microgravity remains unknown. Given the role of Hfq as a regulator of virulence genes in both animal pathogens and phytopathogens, the regulon’s differential expression in human pathogens in spaceflight might also confer increased virulency in plant hosts as demonstrated in animal hosts. Taken together, there exists an alarming knowledge gap regarding plant interactions with human foodborne pathogens in microgravity, as well as interactions with bacteria in general. This is pertinent as foodborne bacterial pathogens have already been unintentionally introduced to space vehicles and space-grown lettuce has displayed colonization by a diverse microbiome. Additional studies using both MMA platforms and spaceflight to study crop plants inoculated with common foodborne pathogens such as *Salmonella enterica* and *E. coli* are necessary to ensure human safety during space travel. A One Health approach to this challenge, connecting the human crew, the spaceflight microbiome, and plants, is needed to ensure our safety as we continue to spend longer periods in spaceflight habitats.

## Data Availability

This review is a synthesis of the findings of others which are readily accessible via peer-reviewed academic journals and academic/government repositories. The references section of this manuscript contains the necessary information to access cited literature. Figure 2 contains micrographs from our unpublished work displaying qualitative phenotypes.
